# Unravelling the Enigma of Scrub Typhus: A Critical Review and Insights Into Epidemiology, Clinical Features, Diagnostic Advances, and Emerging Trends

**DOI:** 10.7759/cureus.62867

**Published:** 2024-06-21

**Authors:** Mrunali S Tarale, Anita B Sajjanar

**Affiliations:** 1 Microbiology, Jawaharlal Nehru Medical College, Datta Meghe Institute of Higher Education and Research, Wardha, IND; 2 Pathology, Datta Meghe Medical College, Nagpur, IND

**Keywords:** immunofluorescence antibody (ifa), weil felix test, orientia tsutsugamushi, zoonotic disease, rickettsial infection, scrub typhus

## Abstract

Scrub typhus (ST) is caused by the bacterium *Orientia tsutsugamushi*, which exhibits significant antigenic diversity and is prevalent in the Asia-Pacific region. Its clinical presentation is characterized by non-specific symptoms such as headache, myalgia, sweating, and vomiting, along with the abrupt onset of fever and chills. An eschar is often visible in the axilla, groin, or inguinal area and is present in around half of the confirmed cases. The Weil-Felix test is the earliest detection technique, though it is not highly specific. Diagnostic procedures include biopsy, culture, serology, and PCR. The molecularly detectable amount of *Orientiae *in the bloodstream occasionally reaches levels that are identified during acute illness and vanish after the first round of appropriate antibiotic treatment. This study offers a thorough review of ST, a disease carried by vectors caused by *Orientia tsutsugamushi.* We emphasize on the significance of monitoring and awareness campaigns, diagnostic problems, and geographical differences. It is essential to use multidisciplinary techniques combining epidemiologists, public health authorities, and doctors. Sustained observation and research are essential for developing successful preventative and control measures. When participating in outdoor activities in areas known for such infections or at particular times of the year when ticks or mites carry the rickettsia infection, people should take great precautions to prevent getting bitten by ticks or mites. Prompt medical evaluation is advised if suspicious symptoms or signs appear, especially in elderly individuals.

## Introduction and background

Epidemiology and clinical features

In the recent years, scrub typhus (ST) has become the most prevalent rickettsial disease in India [1]. In years 2011-2021, patients had a death rate as high as 38.9%, with an overall case-fatality rate of 6.3%. Significant morbidity is responsible for a substantial proportion of fatalities [2]. *Orientia tsutsugamushi *is an obligate intracellular bacterium (earlier called *Rickettsia tsutsugamushi*). *R. tsutsugamushi *and other species of the genus Rickettsia were recently placed on a phylogenetic tree that includes 38 species of *Proteobacteria *based on analysis of the 16S rRNA sequence of *R. tsutsugamushi* [3]. In the new classification, the genus *Rickettsia *now includes 31 species, and new species are introduced to the genus annually [4]. The genus *Rickettsia* that causes rickettsioses, *Orientia* spp., which causes scrub typhus, and members of the Anaplasmataceae family, which cause ehrlichiosis and anaplasmosis, are among the bacteria in the order Rickettsiales that cause illnesses [5]. ST occurs following the bite of an infected trombiculid mite. Fevers, rashes, eschars, meningitis, pneumonitis, and, in certain cases, disseminated intravascular coagulation that causes circulatory collapse are seen [6].

Geographical distribution

Apart from the "Tsutsugamushi triangle" in the Asia-Pacific where it usually occurs, scrub typhus may have a broader worldwide distribution in tropical and subtropical areas, according to findings from the Arabian Peninsula, Chile, and potentially Kenya [7-10]. In the Asia-Pacific region, which includes but is not limited to China, India, Indonesia, Japan, Korea, Taiwan, Sri Lanka, and Thailand, ST is a severe public health concern [11]. In India, numerous cases have been reported from Maharashtra and Rajasthan in the West; Meghalaya, Assam, and Nagaland in the Northeast; Tamil Nadu, Andhra Pradesh, Karnataka, and Kerala in the South; Himachal Pradesh, Uttarakhand, and Jammu & Kashmir in the North; and West Bengal and Bihar in the East.

Diagnostic methods

The current gold standard reference diagnostic method is indirect immunofluorescence assay (IFA) but is not used regularly. PCR assays are used to detect the *Orientia* DNA before antibody responses occur and have a diagnostic advantage in endemic areas with high background levels of antibody in the population. In a study, de-roofed eschar samples were collected and stored in absolute alcohol at -70°C until DNA extraction. Patients with a clinical illness compatible with scrub typhus, including an eschar and positive results for serum samples testing by IgM ELISA, were included. The PCR molecular test was done for further identification [12-13]. When given the right care, most ST patients fully recover from their illness. Clinical features of ST patients are similar to those of other diseases, including typhoid, dengue fever, malaria, leptospirosis, and other feverish conditions. A final diagnosis of ST is made based on the patient's medical history, physical examination, thorough description of their clinical characteristics, and trustworthy diagnostic procedures because these diseases share many symptoms [14]. On the other hand, serious complications and even mortality might arise from a delayed diagnosis and inappropriate care of the ST patient. The level of ​​​​​​*Orientiae* in the bloodstream (fourfold rise to 1:200) that can be shown by molecular methods sporadically reaches levels detected during acute infection, and disappears following the first round of adequate antibiotic therapy. Antibodies do not reach detectable levels until 5 to 10 days after disease presentation. Unfortunately, the preferred specimen, a biopsy of the eschar, is seldom acquired. However, the amount of* Orientia* DNA present in the lesion is abundant, unaffected by previous antibiotic treatment, and remains there for the duration of the disease [15-17]. Biopsy and culture required for the diagnosis are time-consuming procedures. The earliest test used is the Weil-Felix OX-K agglutination reaction, which is inexpensive, easy to perform, and results are available overnight; however, it lacks specificity and sensitivity. Even with its four-week average turnaround time for identification, the culture approach is risky and the test is not available in most laboratories. While the sensitivity has been shown to range from 45% to 82%, 16S rRNA and groEL gene RT-PCR exhibit good specificity [18-20]. Both traditional polymerase chain reaction and the loop-mediated isothermal amplification (LAMP) tests are less effective than qPCR. qPCR assays show a better sensitivity ranging from 45% to 82%. One crucial feature in the diagnosis of rickettsialpox or scrub typhus has been recognised as eschar [21]. It has been discovered that *Orientia tsutsugamushi* may be effectively identified as rickettsialpox in paraffin-embedded skin biopsy tissues by using anti-*Rickettsia rickettsii *antibody immunohistochemical labelling [22].

## Review

Prevalence, clinical manifestation, and diagnosis of scrub typhus in India

Prevalence and Clinical Manifestation

Vanramliana et al. analyzed data from 2018 to 2022, reporting 19,651 cases of scrub typhus out of 22,914 cases of rickettsial illnesses. Aizawl had the highest number of cases [1]. Narang et al. found scrub typhus affecting 15.89% of the population in 2023, with a higher prevalence among individuals aged 41-60, females, and farm workers, particularly during monsoon and post-monsoon seasons [22]. Pandey et al. highlighted a significant number of cases in Eastern India, especially Nagaland and Manipur [23]. Devamani et al. conducted a study in south India, in the Tamil Nadu districts of Vellore, Thiruvannamalai, and Salem, and recruited 1,353 individuals from 15 peri-forest hill villages, 17 rural plain villages, and 16 urban communities. Of them, 71% were older than 30 years and 63% were female; 28.1% of the participants had scrub typhus [24].

Diagnosis

Damodar et al. identified scrub typhus as a cause of acute encephalitis syndrome (AES), emphasizing the use of diagnostic methods like IgM ELISA, and considered an optical density (OD) cutoff of 0.8 in serum and 0.5 in CSF samples to be positive. Scrub typhus was diagnosed by ELISA IgM and real-time PCR testing [25]. In a study by Panigrahi et al., 74 (43.5%) out of 170 acute undifferentiated febrile illness (AUFI) patients were diagnosed scrub typhus by IgM ELISA; 44 (or 59% of the total) were male, with a male-to-female ratio of 1.46:1. The age of the patients varied from 29 days to 84 years. The age group of 1-14 years constituted majority of the cases (58.1%) and the >71-year age group constituted the least with 2 (2.8%) cases [26]. Various studies have used different diagnostic techniques such as ELISA, PCR, Weil-Felix test, and rapid diagnostic tests to confirm ST cases.

Clinical Features

Paulraj et al. observed multi-organ involvement and mortality rates associated with scrub typhus [27]. Patricia et al. noted common symptoms of fever of more than five days, with an eschar, rash lymphadenopathy or fever for which the cause was not known, myalgia, and headache [28]. Varghese et al. found clinical features such as acute respiratory distress syndrome (ARDS), shock requiring inotropic support, hepatitis, meningoencephalitis, renal failure, invasive ventilation, and CNS dysfunction [29].

Geographical Distribution

Studies by Varghese et al. highlighted the distribution of cases across different regions of southern India; apart from Vellore, the highest prevalence was found in other *taluks* such as Gudiyatham (51 cases), Polur (44 cases), and Walajah (44 cases) in Tamil Nadu, and Chittoor (123 cases), Tirupati (30 cases), and Porumanilla (15 cases) in Andhra Pradesh. Cases reported from Andhra Pradesh appeared to cluster around the state border with Tamil Nadu (16 taluks) and a few from Peddapuram. Cases were also reported from Nizamabad in Telangana. Studies from India have reported a post-monsoon or winter spike in the number of scrub typhus cases. Factors that are responsible for the increase in the number of cases are housing conditions and local vegetation around the house [30-31].

Diagnostic Challenges and Testing Methods

**Figure 1:**
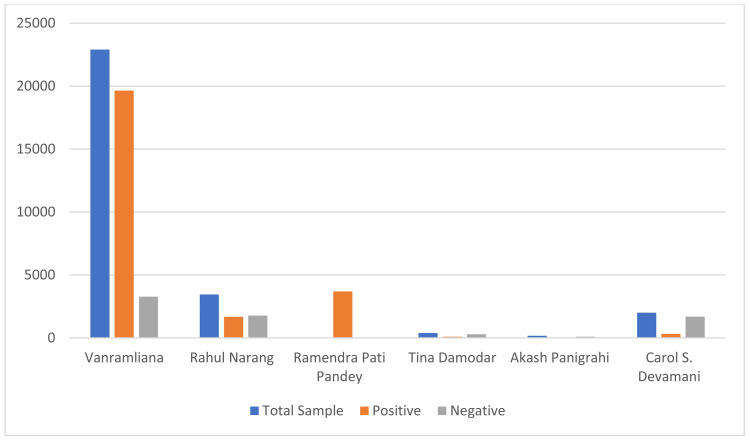
Graphical representation of the studies on the epidemiology of scrub typhus in India Studies by Vanramliana et al. [1], Narang et al. [22], Pandey et al. [23], Damodar et al. [25], Panigrahi et al. [26], and Devamani et al. [24] Image credit: Mrunali Tarale

Anitharaj et al. and Roopa et al. assessed the sensitivity and specificity of diagnostic tests like ELISA (100% and 94.12%) and Weil-Felix (50.38% and 95.51%) against a new IgM immunochromatography test kit [31-33]. This new test kit showed good sensitivity and specificity. Overall, these studies contribute to our understanding of the epidemiology, clinical presentation, and diagnostic approaches for scrub typhus in India, as shown in Figure 1 and Table 1.

**Table 1:** Studies on the epidemiology of scrub typhus in India ICT, immunochromatographic test; IFA, immunofluorescence assay; WFT, Weil–Felix test

Author	Year	Total sample	Positive	Negative	Test
Vanramliana et al. [1]	2023	22,914	19,651 (85.75%)	3263 (14.24%)	WFT, ICT
Narang et al. [22]	2023	3454	1682 (48.69%)	1774 (51.36%)	IgM ELISA IFA
Pandey et al. [23]	2023	-	3697	-	ICT, PCR, WFT, ELISA
Damodar et al. [25]	2023	376	87 (23.13%)	289 (76.86%)	IgM ELISA, RT-PCR
Panigrahi et al. [26]	2023	170	74 (43.52%)	96 (56.47%)	ELISA, ICT
Devamani et al. [24]	2022	2002	314 (15.68%)	1688 (84.31%)	IgG ELISA

Prevalence, clinical manifestation, and diagnosis of scrub typhus in other countries

Diagnostic Challenges and Testing Methods

IgM ELISA has a high sensitivity (96.77%) and specificity, making it a reliable tool for diagnosis of scrub typhus. In contrast, the Weil-Felix test has a lower sensitivity (69.76%) and it may be cross-reactive to other species and may also give false positive results. Tran et al. highlighted the diagnostic challenges and methods used for scrub typhus. They found positive results in 45 out of 114 suspected cases using PCR and ELISA testing [33]. Kinoshita et al. emphasized the difficulty in detecting *Orientiae* in the bloodstream during acute illness and the importance of molecular methods for diagnosis [34].

Epidemiological Trends

Li et al. observed a significant increase in scrub typhus cases in China, with an annual incidence rate climbing from 0.09 to 1.60 per 100,000 population from 2006 to 2016. The ST incidence follows a seasonal pattern that shows a spike in May, a peak in October, and a decline in November; in both the 1980-1989 and the 2006-2016 periods, 94% of cases occurred during this time [35]. Lamichhane et al. reported a high seroprevalence of scrub typhus among AUFI patients in Nepal, with a case fatality rate of 2.56% [36].

Clinical Characteristics and Outcomes

Won et al. identified confirmed cases of scrub typhus using real-time PCR, with various clinical presentations observed [37]. Alam et al. found that the most common clinical features were altered sensorium, fever, headache, myalgia, and the variable presence of eschar of ST-associated encephalitis syndromes, with a pooled case fatality rate ranging from 1.5% to 6.4% [38].

Risk Factors and Exposure

Tasak et al. identified factors associated with scrub typhus exposure among hill tribe members due to cutting grass; these included not using protective clothing or gear for farming, age (above 18 years), education level (poorly educated), tribe affiliation (Akha, Lahu, Hmong, Yao and Lisu tribes), and occupational activities (agricultural work) [39]. These studies provide insights into the epidemiology, clinical manifestations, diagnostic approaches, and risk factors associated with scrub typhus in various countries as depicted in Table 2.

**Table 2:** Studies on the epidemiology of scrub typhus in other countries RDT, rapid diagnostic kit; IFA, immunofluorescent assay; WFT, Weil-Felix test

Author	Country	Year	Total sample	Positive	Negative	Tests
Tran et al. [33]	Switzerland	2021	114	44 (38.59%)	70 (61.40%)	PCR buffy coat and/or ELISA IgM
Li et al. [35]	China	2020	133,623	-	-	-
Lamichhane et al. [36]	Nepal	2023	10,977	2120 (19.31%)	8857 (80.68%)	ELISA, RDT, IFA, WFT
Won et al. [37]	South Korea	2023	187	37 (19.78%)	150 (80.21%)	-
Alam et al. [38]	UK	2022	1221	-	-	-
Tasak et al. [39]	Thailand	2023	485	237 (48.86%)	248 (51.13%)	ELISA, IFA

Discussion

Epidemiology and Regional Variance

Several studies highlight the burden and regional variations of scrub typhus.* *Vanramilana et al. found a high burden in Aizawl, with a 0.35% case fatality rate and 3.54 cases per 1000 person-years. Risk factors for mortality include eschar presence, occupational exposure, and young age (children) [1]. Narang et al. observed 15.80% prevalence, with higher rates in the 41-60 year age group, in females, and among farm workers during monsoon seasons [22]. In the study in Northeast India by Pandey et al., 55.5% of cases were primarily from Nagaland and Manipur, with a 2.4% case fatality rate [23]. Damodar et al. linked scrub typhus to AES in south Indian states [25]. Panigrahi et al. explored acute undifferentiated febrile illness cases, noting 43.5% positivity and higher prevalence among the 1-14 age group [26]. Devamani et al. explored seroprevalence, noting variations across rural-urban and plain regions [24]. Kannan et al. demonstrated a 50% prevalence, highlighting the importance of comprehensive diagnostics [40]. Paulraj et al. focused on specific tribes in Tamil Nadu, reporting a 6.07% positivity rate [27].

Clinical Presentation and Complications

Studies emphasize multi-organ involvement and the need for thorough diagnostics. Devasagayam et al. recorded a 6.3% overall case-fatality rate. Common complications were acute respiratory distress syndrome (ARDS), acute kidney injury (AKI), hepatitis, shock, myocarditis, meningitis, and thrombocytopenia, with higher rates in multi-organ dysfunction syndrome (MODS) patients [2]. Saluja et al. emphasized the need for a comprehensive diagnostic approach [41]. Narlawar et al. explored cases in central India; the common symptoms were fever, fever with chills, breathlessness, cough, altered sensorium, vomiting and nausea, swelling over feet, headache, gastrointestinal symptoms, urinary symptoms, skin lesions (eschar). Other complications are pancreatitis, ARDS, septicemia, encephalitis, AKI, osteoarthritis, and renal calculi [42]. Varghese et al. highlighted case distribution in south India where ARDS, shock requiring inotropic support, hepatitis, meningoencephalitis, renal failure, invasive ventilation and CNS dysfunction were seen, confirming with IgM ELISA [30].

Temporal Trends and Global Perspectives

Tran et al. provided insights into global diagnostic approaches. Clinical examinations, PCR and ELISA IgM tests were used to detect the organism. Among people living in different communes, always observing mice around home, and workplace environment with risk were associated with an increased ST risk. Changing clothes when at home was likely to protect from ST. Among persons in same commune, sitting/laying directly on the household floor was a risk factor [33]. Kinoshita et al. discussed challenges in detecting *Orientiae* and obtaining detectable antibody levels [34]. Li et al. presented long-term trends in China by analyzing the incidence and spatial-temporal distribution of scrub typhus in China during 1952-1989 and 2006-2016 using national disease surveillance data. A total of 133,623 cases and 174 deaths were recorded. The average annual incidence was 0.13 cases/100,000 population during 1952-1989; the incidence increased sharply from 0.09/100,000 population in 2006 to 1.60/100,000 population in 2016 [35]. Lamichhane et al. investigated the situation in Nepal. The seroprevalence of scrub typhus among the AUFI cases in Nepal was 19.31%. The seroprevalence of scrub typhus among AUFI cases ranged from 5.37% to 40.32% in Nepal [36]. Alam et al. examined cases in the UK, focusing on clinical features of scrub typhus-associated encephalitis syndromes [38]. They concluded that clinicians should have a high index of suspicion for ST in patients presenting with AES.

Challenges and future considerations

Collectively, the studies included here highlight challenges in diagnosis, regional variations, and the importance of surveillance and awareness programs. Multidisciplinary approaches involving clinicians, epidemiologists, and public health officials are crucial. Continued research and surveillance are necessary for effective prevention and control strategies.

## Conclusions

We used recent surveillance data to define the seasonal and geographic characteristics of scrub typhus. This background data may be beneficial to people involved in the medical field and and public health. This knowledge can be useful in the decision-making process when identifying people who may have vector-borne rickettsial infections because the two rickettsial illnesses are difficult to distinguish based on clinical symptoms. People should make serious efforts to avoid getting bitten by mites or ticks while participating in outdoor activities, in endemic regions, or during specific periods of the year when the mites or ticks are known to harbour the rickettsia virus. Following such exercise, a quick medical evaluation should be sought when questionable symptoms or signs emerge, especially in seniors.
